# Awake Implementation of Extracorporeal Life Support in Refractory Cardiogenic Shock

**DOI:** 10.3390/medicina58010043

**Published:** 2021-12-28

**Authors:** Julia Riebandt, Thomas Haberl, Klaus Distelmaier, Martin H. Bernardi, Anne-Kristin Schaefer, Guenther Laufer, Daniel Zimpfer, Dominik Wiedemann

**Affiliations:** 1 Division of Cardiac Surgery, Medical University of Vienna, 1090 Vienna, Austria; thomas.haberl@meduniwien.ac.at (T.H.); anne-kristin.schaefer@meduniwien.ac.at (A.-K.S.); Guenther.laufer@medunwien.ac.at (G.L.); daniel.zimpfer@meduniwien.ac.at (D.Z.); dominik.wiedemann@meduniwien.ac.at (D.W.); 2 Division of Cardiology, Medical University of Vienna, 1090 Vienna, Austria; klaus.distelmaier@meduniwien.ac.at; 3 Division of Cardiac Thoracic Vascular Anesthesia and Intensive Care Medicine, Medical University of Vienna, 1090 Vienna, Austria; martin.bernardi@meduniwien.ac.at

**Keywords:** cardiogenic shock, ECLS, awake implantation, techniques

## Abstract

*Background and objectives:* Extracorporeal life support (ECLS) is a widely accepted and effective strategy for use in patients presenting with refractory cardiogenic shock. Implantation in awake and non-intubated patients allows for optimized evaluation of further therapy options while avoiding potential side effects associated with the need for sedation and intubation. The aim of the study was the assessment of safety and feasibility of awake ECLS implementation and of outcomes in patients treated with this concept. *Materials and Methods:* We retrospectively reviewed the concept of awake ECLS implantation in 16 consecutive patients (mean age 58 ± 8 years; male: 88%; ischemic cardiomyopathy: 50%) from 02/2017 to 01/2021. Study endpoints were survival to weaning or bridging to durable support or organ replacement and development of end-organ function and hemodynamic parameters on ECLS. *Results:* Fourteen patients (88%) were able to be successfully transitioned to definite therapy options. ECLS support stabilized end-organ function, led to a decrease in mean lactate levels (5.3 ± 3.7 mmol/L at baseline to 1.9 ± 1.3 mmol/L 12 h after ECLS start; *p* = 0.01) and improved hemodynamics (median central venous pressure 20 ± 5 mmHg vs. 10 ± 2 mmHg, *p* = 0.001) over a median duration of two days (1–8 days IQR). Two patients (13%) died on ECLS support due to multi-organ dysfunction syndrome. Survival to discharge of initially successfully bridged or weaned patients was 64%. *Conclusions:* Awake ECLS implantation is feasible and safe with the key advantage of omitting or delaying general anesthesia and intubation, with their associated risks in cardiogenic-shock patients, facilitating further decision making.

## 1. Introduction

Despite all advances in medical management and the broad use of mechanical circulatory support (MCS), refractory cardiogenic shock (RCS) is still associated with a high mortality rate of up to 67% [1,2,3]. Regarding the underlying disease and pathomechanisms, patients are inhomogeneous, but all present with signs of end-organ deterioration (renal replacement in over 30%), elevated lactate levels and need for one or more vasoactive agent to maintain adequate systemic blood pressure or often even MCS implantation (nearly 50%) [3].

Even though extracorporeal life support (ECLS) has been shown to effectively provide emergent biventricular circulatory and respiratory support, there is also a number of disadvantages, including a limited duration of support, a high rate of thromboembolic events, risk of bleeding and infection, with sepsis and multiple organ dysfunction syndrome being the major cause of death during ECLS support [4,5,6]. The risk of experiencing one of these complications increases with duration of ECLS; therefore, patients should be evaluated for further therapy as soon as possible [7].

Usually, patients in cardiogenic shock requiring ECLS support are under general anesthesia, intubated and on high-vasopressor support or they will be sedated and intubated before implantation of ECLS. This may put the patient at risk for multiple complications; the sedation and intubation process in hemodynamic highly unstable patients, per se, brings the risk of immediate right ventricular decompensation and can result in resuscitation [8]. However, not only is the process itself risky; additionally, even when ECLS implantation is successful, the following can be observed: greater need for vasopressor support (bradycardia and hypotension are well-recognized side effects of general anesthesia, and cardiac output is also decreased); muscular atrophy and venous embolism due to sedation and immobilization; risk for ventilator-associated lung injury; pneumonia or interference with right atrial filling; and venous return due to intrathoracic pressure changes [8,9,10]. Furthermore, further therapy decisions necessitating neurological evaluation might be delayed in sedated and intubated patients, increasing the risk of experiencing ECLS complications due to longer running times.

We present a series of patients in whom the concept of awake ECLS implementation with omittance or delay of general anesthesia and intubation was followed, allowing for further therapy evaluation or weaning in an optimized setting while reducing the risk inherent to sedation and intubation of patients presenting with critical cardiogenic shock.

## 2. Materials and Methods

### 2.1. Study Population

From 02/20017 to 01/2021, 16 non-intubated patients in RCS received temporary mechanical circulatory support, using the awake ECLS implantation concept. Overall adult ECLS volume within the observation period was 663 (including 119 patients in cardiogenic shock). Data were obtained retrospectively, and the study was approved by the Ethics Committee of the Medical University of Vienna (1440/2020).

### 2.2. Indications and Conduct of ECLS

The decision for ECLS implantation was made by an interdisciplinary team consisting of at least one cardiac surgeon and one cardiologist or anesthesiologist/intensivist. Indication for temporary support was presence of cardiogenic shock stage D and E, as described by Baran et al. in their consensus paper regarding the classification of cardiogenic shock [11]. ECLS implantation was only done in patients with any kind of further treatment option: bridge to potential organ-preserving procedure (e.g., percutaneous coronary intervention, surgical ventricular septum defect closure, etc.) or bridge to durable assist device or heart transplantation.

The technique has been described previously by our group, including a video of the procedure [12]. Patients are placed in a supine position but allowed to lie at an incline of 30–40 degrees to facilitate breathing. Oxygen insufflation is provided via a mask. A radial artery line for continuous monitoring and blood-gas retrieval is installed if not already present. Local anesthesia using xylocaine 2% is administered, and then both femoral vessels are punctured approximately two centimeters below the inguinal ligament, and guidewires are inserted under transthoracic echocardiography or abdominal sonography control (depending on availability on location of ECLS implantation). A quantity of 5000 units of unfractionated heparin is given systemically before continuing with dilation of the vessels and insertion of the cannulas (arterial: 17–19 French, venous: 19–21 French) according to the Seldinger technique. If possible, a distal leg perfusion canula (6 French) is implanted in the same session. In any case, oxygen saturation of both legs is monitored using near-infrared spectroscopy.

The patient is under close anesthesiologic monitoring during the procedure, and a light sedation using midazolam can be administered, if necessary, still guaranteeing sufficient spontaneous breathing.

ECLS is started once the cannulas are connected. Flow setting is guided by hemodynamics and should allow for at least intermittent aortic valve opening to promote left ventricular unloading and washout.

As per protocol, patients on ECLS get a daily routine lab, as well as controls of anticoagulation and hemolysis status and arterial blood-gas samples three times per day. Anticoagulation during the ECLS run is achieved with unfractionated heparin monitored by activated partial thromboplastin time (aPTT), with a target therapeutic aPTT of 2–2.5× baseline. In case of confirmed or suspected heparin-induced thrombocytopenia, argatroban is used instead of heparin, with the same target aPTT.

As ECLS running times should be kept as short as possible to avoid complications, evaluation for potential cardiac recovery via transthoracic or transesophageal echocardiography (LVEF, aortic VTI, lateral mitral annulus systolic peak velocity, RV Dilatation/new onset of severe tricuspid insufficiency, RV FAC, lateral tricuspid annulus peak velocity) is performed on a regular basis, starting 24–48 h from ECLS implantation, using a standardized institutional weaning protocol developed by our intensivists over the last years. It contains three key aspects: (1) recovered hemodynamical situation (less than 3 L/min blood flow, pulsatile arterial wave form for at least 24 h and no dependence on high-dose vasopressor or inotrope support); (2) respiratory stability (FiO_2_ (ECLS) < 0.5; FiO_2_ (ventilator) < 0.6; minute ventilation > 6 L/min; ECLS gas flow < 2 L/min) and (3) End-organ recovery.

Explantation of the cannulas is usually performed via surgical cutdown, followed by thrombectomy of the femoral artery using Fogarty catheters to reduce the risk for embolism originating from the arterial cannula.

### 2.3. Outcome Measures

Primary endpoint was survival to weaning or bridging to durable support or organ replacement. Secondary endpoints were (1) development of end-organ function on ECLS, (2) development of hemodynamic parameters and of catecholamine support and (3) survival after bridging.

### 2.4. Statistical Analysis

Categorical variables were presented as numbers and percentages, and mean values and standard deviations (SD) or median with range were determined for continuous variables. Comparison of means was performed using the paired t-test or Wilcoxon test, when appropriate. A *p*-value < 0.05 was considered statistically significant. Survival was determined using the Kaplan-Meier method.

IBM SPSS software version 26 (SPSS, Inc., Chicago, IL, USA) was used for statistical analysis.

## 3. Results

### 3.1. Patient Characteristics

Sixteen non-intubated patients presenting with RCS underwent ECLS implantation without sedation and intubation between 02/2017 and 01/2021. Mean age was 58 ± 8 years, and 88% were male. The underlying disease was an ischemic cardiomyopathy (ICMP) in eight patients (50%), with one suffering from an ischemic VSD, dilatative cardiomyopathy (DCMP) in five patients (31%), two patients with myocarditis (one of them COVID-19-associated) and one patient with amyloidosis. With the exception of one patient, all were transferred from external intensive care units without the possibility of advanced surgical heart failure therapies. Seven patients (44%) were already in renal failure, necessitating continuous renal replacement therapy, and most patients (88%) showed signs of decreased liver function and venous congestion with hyperbilirubinemia and elevated transaminases. The majority (94%) required hemodynamic support with at least two vasoactive agents at the time of ECLS implementation. Immediately before implantation, the mean calculated survival after venoarterial ECMO (SAVE Score) was −6.1 ± 5.8, correlating with a predicted survival to discharge of only 30% [13].

### 3.2. ECLS Settings

Initial ECLS flow rates showed a median of 2.7 (2.5–3 IQR) L/min. They were adapted according to hemodynamics but should only give partial support to allow for aortic valve opening, as well as left ventricular washout. There was only one patient requiring full support, who received a left ventricular vent later on. Respiratory support was provided with a mean gas flow of 2 ± 1.4 L and a median fraction of inspired oxygen (FiO_2_) of 0.6 (0.6–1.0).

### 3.3. Development of End-Organ Function during ECMO

#### 3.3.1. Renal Function

All patients presented with signs of renal dysfunction before ECLS implementation (mean creatinine 1.90 ± 0.82 mg/dL; mean BUN 51.32 ± 28.82 mg/dL). Nearly half of the patients (44%) were already depended on venovenous hemofiltration, and ECLS did not change laboratory values of renal function over a median duration of two days of support (range 1–10). However, with the exception of one patient listed for combined heart and kidney transplantation, renal function fully recovered in all surviving patients, and none remained dialysis-dependent after ICU discharge.

#### 3.3.2. Hepatic Function

Most patients had elevated liver parameters. ECLS led to right ventricular unloading and improved hepatic function in all patients (median ASAT 174 (66-1316) U/L pre ECLS vs. 94 (39-260) U/L post ECLS, *p* = 0.009; median ALAT 171 (30-906) U/L pre ECLS vs. 64 (34-277) U/L, *p* = 0.003) over a median duration of two days of support (range 1–10).

### 3.4. Development of Hemodynamic Parameters and Catecholamine Support during ECLS

Neither extensive echocardiographic examinations nor hemodynamic monitoring via Swan-Ganz catheter was feasible in the acute setting. Hemodynamic evaluation pre- and post-ECLS was therefore limited to measurement of the central venous pressure (CVP), mean arterial pressure (MAP), heart rate (HR), the central venous oxygen saturation (ScvO2) and lactate serum levels.

When comparing pre-ECLS data to the values after ECLS initiation, a strong tendency of improved hemodynamics was observed (mean central venous pressure 20 ± 5 mmHg vs. 10 ± 2 mmHg, *p* = 0.008; mean heart rate 122 ± 13 bpm vs. 97 ± 13 bpm, *p* = 0.000; median central venous oxygen saturation 42 (40–65) % vs. 80 (60–84) %, *p* = 0.317; mean arterial pressure 68 ± 12 mmHg vs. 74 ± 8 mmHg, *p* = 0.194), though the latter are not statistically significant due to the small number of patients.

A significant decrease in lactate serum levels (5.3 ± 3.7 mmol/L at baseline to 1.9 ± 1.3 mmol/L, *p* = 0.001) was already observed after the first 12 h of the ECLS run, as well as a lower vasoactive inotropic score (VIS; 51 (16–284) vs. 19 (8–94), *p* = 0.015), mostly due to a reduction in vasopressor support. Inotropic support remained nearly the same over the ECLS course, supporting myocardial contractility and facilitating aortic valve opening, as well as ventricular washout. All assessed clinical parameters are depicted in Table 1.

### 3.5. Outcomes

#### 3.5.1. Outcome while on ECLS Support

All ECLS implantation procedures were uneventful; no switching to standard implantation techniques requiring sedation and intubation or even surgical cutdown was necessary. Median ECLS duration was two days (1–10 days). Fourteen patients (86%) were able to be successfully bridged (durable assist device: *n* = 8; HTX: *n* = 3; weaning after myocarditis recovery: *n* = 2; weaning after surgical closure of an infarct VSD: *n* = 1). Two patients died (POD 2 and POD 10) of multiorgan dysfunction syndrome.

During ECLS support, we did not observe ECLS-related complications, such as bleeding, thromboembolism or cerebrovascular events. Only one patient needed blood-product substitution due to further deterioration of liver function, leading to a general bleeding disorder. However, we observed a probable ECLS-explantation-related limb ischemia and femoral artery bleeding necessitating intervention a few days after ECLS explantation in one patient, respectively, none with permanent damage. Six patients required intubation several hours after ECLS implantation and hemodynamic stabilization. Two of them did not meet the primary endpoint of successful weaning or bridging. The other four were brought to definitive surgical therapy within a few hours after intubation. The most common reasons for secondary intubation were agitation or respiratory failure.

Importantly, the safe run of ECLS was not compromised by the fact that patients were awake and actively mobilized; no canula dislodgement, air embolism or similar complications were observed.

#### 3.5.2. Outcome after Successful Bridging to Durable Assist Devices

Seven patients underwent successful LVAD implantation (less invasive technique in 38%, as described previously by our group) after a median ECLS duration of one day (1–3 days), without intraoperative transition to cardiopulmonary bypass in all but one patient, who needed a modified Park’s stitch due to moderate aortic regurgitation [14,15]. All patients stayed on postoperative right ventricular support for a mean duration of 11 ± 9 days, and they were transitioned to a temporary RVAD with graft-facilitated canulation of the main pulmonary artery. Two patients required surgical re-exploration due to hemothoraces on postoperative day 1. In one patient, the bleeding source was able to be identified (lung injury from chest-tube placement a few days earlier in a peripheral hospital, which became relevant after anticoagulation start); the other patient presented with diffuse bleeding from over-anticoagulation. One patient suffered a perioperative non-disabling ischemic stroke but nearly fully recovered with physiotherapy. Otherwise, no further bleeding or thromboembolic complications occurred in the perioperative phase.

Three of these patients died from multiorgan dysfunction syndrome during the index hospital stay on postoperative day 256, 254 and 246, respectively.

One patient suffered severe biventricular failure and was not suitable for transplantation; he underwent implantation of a total artificial heart but, unfortunately, developed fatal intracerebral bleeding in the perioperative phase.

#### 3.5.3. Outcome after Successful Bridging to Heart Transplantation

Three patients already on the transplantation waiting list before developing cardiogenic shock were switched to international high-urgency status and were successfully transplanted. However, one patient developed severe multiorgan failure and died on postoperative day 30.

#### 3.5.4. Outcome after Successful Weaning

Two patients with myocarditis (one of them COVID-19-related) and one patient suffering from ischemic VSD after acute myocardial infarction who underwent surgical VSD closure after two days of the ECLS run were able to be successfully weaned without complications after a duration of 6 ± 4 days. After a median follow-up time of 53 days, all three patients were still alive and in ambulatory care.

#### 3.5.5. Overall Hospital Stay and Survival Rates

Median duration of ICU stay was 20 (18–83 IQR) days; median length of hospital stay was 45 (26–128 IQR) days.

**Figure 1:**
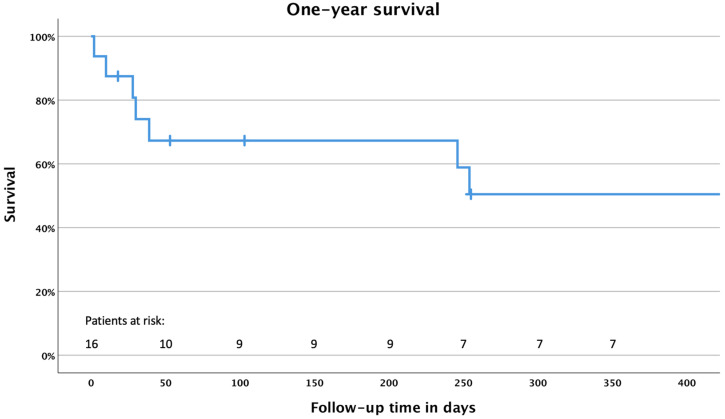
Kaplan-Meier analysis of the 1-year survival.

Thirty-day mortality was 7%; in-hospital mortality 36%. The estimated one-year survival of 64% is depicted in Figure 1.

## 4. Discussion

Refractory cardiogenic shock is a devastating condition that is associated with high morbidity and mortality. Despite optimized medical management, mortality rates are still high and patients often need temporary mechanical circulatory support to reduce their initial acuity and stabilize them until further therapy options have been evaluated and decided [1,2,3,16,17].

For ECLS implantation, usually patients are fully sedated and intubated; however, in lung transplant recipients with acute respiratory distress syndrome, awake venovenous extracorporeal membrane oxygenation (ECMO) implantation has been shown to be associated with better results after lung transplantation when compared to traditional ECMO implantation strategies [18].

We present our institutional experience with an awake ECLS implementation approach in cardiogenic shock as an attempt to positively influence outcomes of these patients.

The primary finding of the study is that after successful transition to definite therapies, such as surgical VSD closure, LVAD or HTX, outcomes are excellent, with a 90-day survival of 79%.

Similar to the traditional ECLS implantation technique, patients were able to be rapidly and effectively stabilized within hours after ECLS initiation. Lactate levels a as surrogate parameter for myocardial hypoxemia was able to be significantly lowered in all patients. However, end-organ recovery and hemodynamic stabilization were not achieved permanently in all patients. In this series, two patients (22%) did not meet the primary endpoint of successful bridging, further deteriorated on ECLS and died of multiorgan dysfunction syndrome. This percentage is similar to what is described in the two other studies investigating awake ECLS implantation in nine and 23 patients, respectively [19,20].

In this series, we found a rather low rate of ECLS-related complications; the two events (bleeding and limb ischemia) occurred with a delay of a few days after explantation and are more related to the explantation procedure than to the ECLS itself. Need for secondary intubation occurred in 44% of patients. Upon detailed review of data, we observed—not surprisingly—a greater need for vasopressors for intubated patients. Once again, due to small numbers, a meaningful statistical interpretation is not possible; however, this trend is an important finding, as need for sedation and intubation is associated with pulmonary morbidity, and greater vasopressor support has been shown to negatively influence outcomes in numerous ways, such as an increase in myocardial oxygen consumption and peripheral hypoxemia due to vasoconstriction, with risk for relevant ischemia and necrosis [9,10].

A key advantage of awake ECLS implantation (and to try to keep them awake) is the avoidance of sedation and intubation in patients presenting with therapy refractory cardiogenic shock, as such patients are especially prone to slip into arterial hypotension and ultimately a cardiopulmonary resuscitation (CPR) situation once sedation has to be installed. Since catecholamine dosages are often already at a maximum level and the patient’s cardiac function is highly impaired, success of resuscitation using drugs alone is unlikely. Implanting ECLS under reanimation, on the other hand, is not only technically more challenging but also associated with worse outcomes compared to non-reanimation situations.

Furthermore, awake ECLS patients can communicate in case of pain, for example, arising from limb ischemia or other thromboembolic events; they can drink and eat and avoiding feeding via nasogastric tube; they can participate in physiotherapy and can be mobilized more easily, avoiding loss of muscle during the waiting time until definitive treatment and thereby also improving the outcome of subsequent procedures, such as LVAD implantation or heart transplantation. Apart from that, it is possible to discuss and achieve informed consent for further treatment options, which patients can decide on together with their families. A potentially higher risk for cannula dislodgment after mobilization with the possibility of detrimental complications, such as air embolism, has not been observed in this cohort [21].

## 5. Limitation

The present study has the typical limitations of a retrospective analysis in a small patient cohort. However, the main question of safety and feasibility of the awake ECLS concept, as shown in the number of patients meeting the primary endpoint and the low adverse-event rate, can be sufficiently answered, despite the lack of a control group.

## 6. Conclusions

Awake ECLS implementation in non-intubated patients with refractory cardiogenic shock is safe and feasible. It provides immediate hemodynamic stabilization and effectively leads to end-organ recovery, given the right timing and indication, similar to traditional ECLS implantation techniques. The main advantage of avoidance or deferral of general anesthesia and subsequent intubation is that it might reduce the adverse-event burden in this high-risk patient cohort. Eventually, multicenter studies could give further insights on the potential benefits.

## Figures and Tables

**Table 1:** Clinical parameters pre and post ECLS (*n* = 16).

	Pre-ECLS	Post-ECLS	*p*-Value
Cr (mg/dL)	1.88 ± 0.82	1.83 ± 0.99	0.773
Bilirubin (mg/dL)	2.38 ± 1.93	5.05 ± 4.92	0.091
ASAT (U/L; IQR)	174 (66–1316)	94 (39–260)	**0.009**
ALAT (U/L; IQR)	171 (30–106)	64 (34–277)	**0.003**
Hemoglobin (mg/dL)	11.61 ± 2.39	9.53 ± 1.36	**0.001**
CRP (mg/dL; IQR)	6.34 (4.46–12.92)	7.30 (3.62–16.16)	0.326
Platelets (×109)	205 ± 102	133 ± 60	**0.004**
CVP (mmHg)	20 ± 5	10 ± 2	**0.001**
MAP (mmHg)	68 ± 12	74 ± 8	0.194
HR (bpm)	122 ± 13	97 ± 13	**0.000**
ScvO2 (%; IQR)	42 (40–65)	80 (60–84)	0.317
VIS (points; IQR)	51.5 (16.3–284.0)	13.1 (6.0–77.0)	**0.015**
Lactate (mmol/L)	5.27 ± 3.67	1.87 ± 1.33	**0.001**
MELD-xi-score (IQR)	21 (16–23)	22 (17–28)	0.098

Unless otherwise indicated, data expressed as mean ± SD. Cr: creatinine, ASAT: aspartate aminotransferase, ALAT: alanine aminotransferase, CRP: C-reactive protein, CVP: central venous pressure, MAP: mean arterial pressure, HR: heart rate, ScvO_2_: central venous oxygen saturation, VIS: vasoactive-inotropic score, IQR: interquartile range. Bold: statistically significant results

## Data Availability

The data presented in this study are available on request from the corresponding author. The data are not publicly available due to ethical and legal aspects.
